# Paleogenetic Analyses Reveal Unsuspected Phylogenetic Affinities between Mice and the Extinct *Malpaisomys insularis*, an Endemic Rodent of the Canaries

**DOI:** 10.1371/journal.pone.0031123

**Published:** 2012-02-21

**Authors:** Marie Pagès, Pascale Chevret, Muriel Gros-Balthazard, Sandrine Hughes, Josep Antoni Alcover, Rainer Hutterer, Juan Carlos Rando, Jacques Michaux, Catherine Hänni

**Affiliations:** 1 Institut de Génomique Fonctionnelle de Lyon, Université de Lyon, Université Lyon 1, CNRS UMR 5242, Ecole Normale Supérieure de Lyon, Lyon, France; 2 Institut Mediterrani d'Estudis Avançats, Esporles, Mallorca, Spain; 3 Department of Mammalogy, American Museum of Natural History, New York, New York, United States of America; 4 Zoologisches Forschungsmuseum Alexander Koenig, Bonn, Germany; 5 Departamento de Biología Animal (UDI Zoología), Universidad de La Laguna La Laguna, Tenerife, Canary Islands, Spain; 6 Island Ecology and Evolution Research Group (IPNA-CSIC), La Laguna, Tenerife, Canary Islands, Spain; 7 EPHE–ISEM, UMR 5554 CNRS Université Montpellier II, Université de Montpellier, Montpellier, France; Zoological Society of London, United Kingdom

## Abstract

**Background:**

The lava mouse, *Malpaisomys insularis*, was endemic to the Eastern Canary islands and became extinct at the beginning of the 14^th^ century when the Europeans reached the archipelago. Studies to determine *Malpaisomys'* phylogenetic affinities, based on morphological characters, remained inconclusive because morphological changes experienced by this insular rodent make phylogenetic investigations a real challenge. Over 20 years since its first description, *Malpaisomys'* phylogenetic position remains enigmatic.

**Methodology/Principal Findings:**

In this study, we resolved this issue using molecular characters. Mitochondrial and nuclear markers were successfully amplified from subfossils of three lava mouse samples. Molecular phylogenetic reconstructions revealed, without any ambiguity, unsuspected relationships between *Malpaisomys* and extant mice (genus *Mus*, Murinae). Moreover, through molecular dating we estimated the origin of the *Malpaisomys*/mouse clade at 6.9 Ma, corresponding to the maximal age at which the archipelago was colonised by the *Malpaisomys* ancestor via natural rafting.

**Conclusion/Significance:**

This study reconsiders the derived morphological characters of *Malpaisomys* in light of this unexpected molecular finding. To reconcile molecular and morphological data, we propose to consider *Malpaisomys insularis* as an insular lineage of mouse.

## Introduction

**Figure 1:**
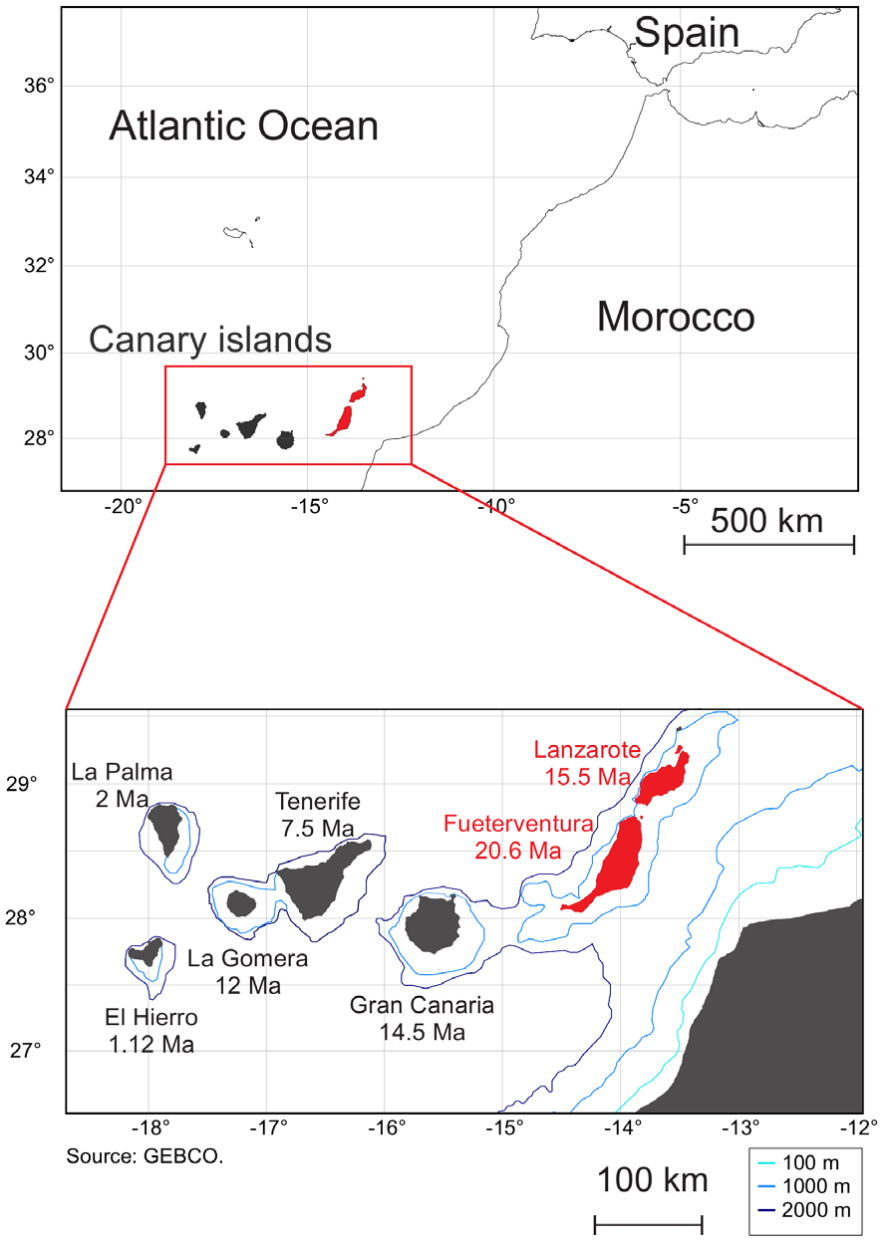
Geographic situation of the Canary Islands. The eastern islands where fossils of *Malpaisomys* have been reported are indicated in red. The K-Ar age of subaerial volcanism is indicated for each island [1]. The maps use the General Bathymetric Chart of the Oceans and were drawn with PanMap [83].

The Canary Islands comprise seven main volcanic islands that appeared successively between 20.6 and 1.12 million years ago (Ma) ([1]; Figure 1). Located ca. 95 km offshore of the Moroccan coast, these islands have been completely isolated from the mainland since their formation. The minimum water depth between them and the continent averages 1,500 m and no connection with the mainland occurred even during the major Pleistocene sea regressions. Current and past biodiversity of the Canaries is thus the result of over-water dispersal events rather than the product of vicariant evolution.

Among the vertebrate endemics, the Canarian shrew, *Crocidura canariensis* is the only living non-flying terrestrial mammal of the archipelago. Three endemic rodents have also been documented since the Quaternary epoch but are nowadays extinct: two giant rats, *Canariomys bravoi* from Tenerife and *Canariomys tamarani* from Gran Canaria [2], plus the lava mouse, *Malpaisomys insularis*, from Fuerteventura, Lanzarote and adjacent islets [3] (Figure 1). The extinction of the genus *Canariomys* seems to be related to the arrival of the aboriginals [4] some time between 756 cal BC and 313 cal AD [5]. *M. insularis* survived until the 14^th^ century [6] and probably became extinct due to the introduction of alien mammals by the Europeans [6], [7]. This study focuses on this latter species.

Until now, using morphological data to determine the phylogenetic affinities of this endemic rodent had not resolved the riddle of its relationship to extant Murinae. *M. insularis* belongs undoubtedly to the subfamily Murinae (Old World Rats and Mice) but its relationships to extant Murinae remained unresolved. There is no fossil evidence to document its morphological evolution from a continental ancestor. Indeed, the earliest remains are evidence of a derived species with uncommon dental and skeletal features [8]. They are consequently difficult to affiliate with certainty to any lineage of Murinae. The first phylogenetic analysis of *M. insularis* was based on twenty dental traits [3]. It included several extinct and extant Murinae from western Europe and Africa: several genera of Arvicanthini, the black rat (*Rattus rattus*), the house mouse (*Mus musculus*), representatives of two genera now shifted from Murinae to Deomyinae, *Acomys* and *Uranomys* [9] and the two other Canarian species (*Canariomys bravoi* and *C. tamarani*). No clear-cut results were obtained except that *Malpaisomys* was grouped with *Acomys* and *Uranomys*. However, this first phylogeny seemed to be flawed by convergent evolution and did not reflect the currently well-established phylogenetic relationships among the Murinae (*e.g.* all the representatives of the tribe Arvicanthini *sensu* Lecompte *et al.* [10] did not cluster together). A few years later, immunological comparisons of albumin proteins extracted from bones of *Malpaisomys* and four other extant rodents (*Mus, Cavia, Acomys* and *Uranomys*) indicated that *Malpaisomys* clustered with *Mus* and was more distant from *Acomys* and *Uranomys* [11]. Although technically innovative, the author only tested the position of *Malpaisomys* within the Murinae (only one murine representative was incorporated into the taxon sampling). The last comparative studies of cranial and dental morphologies included extinct rodents from the late Neogene of western Europe and North Africa to elucidate *Malpaisomys'* relationships [12], [13]. However no conclusion was settled about its systematics. On the basis of these limited data, *Malpaisomys insularis* is currently interpreted as a member of the African tribe Arvicanthini [9].

The collection in 2006 and 2007 of geologically young *Malpaisomys insularis* remains in Fuerteventura [5], [6] combined with ancient DNA studies to tackle new questions on extinct species, gave us the opportunity to clarify the phylogenetic relationships of *Malpaisomys* independently of morphological data. We provide here the first mitochondrial and nuclear DNA sequences for *M. insularis* and perform phylogenetic reconstructions that reveal unsuspected affinities to extant rodents. In return, these new results allow us to re-examine the distinctive morphological features of *M. insularis* in a new evolutionary context.

## Materials and Methods

### Sampling

The native fauna on Fuerteventura and Lanzarote includes two species: the extinct lava mouse, *Malpaisomys insularis* and the extant shrew, *Crocidura canariensis*. Humans introduced alien species, such as the house mouse, *Mus musculus* and the black rat, *Rattus rattus*. Materials excavated from cracks in the volcanic sediments or from sediments filling lava tubes were without ambiguity identified as *Malpaisomys insularis* remains. Their size was higher than expected for shrews or house mice (*e.g.* the femur length is ca 19 mm and 15 mm for *M. insularis*, for *M. musculus* respectively) but smaller than expected for black rats. Moreover, tooth size and molar morphology were unequivocal. The upper and the lower molars were too wide and too stout to belong to *Mus* and on the contrary, too small to be those of *Rattus rattus*. The number of their roots was also too limited and their outlines too wide to correspond to black rat molars. Six fossils of *Malpaisomys insularis*, corresponding to at least four individuals, were analysed in this study (Table 1). To increase the quantity of material used for DNA extraction, 3 half-mandibles were pooled together and were considered further as a single sample (CH475) leading to a total dataset of four samples. Fossils were collected in two different sites from Fuerteventura: Cueva del Llano (in 2006) and Montaña de la Arena (in 2007). The material was obtained through field work within the Research Projects “Cronología y causas de las extinciones de vertebrados autóctonos en Canarias y Baleares: un análisis comparativo. I & II”, both funded by the Dirección General de Investigación of the Spanish Ministerio de Educación y Ciencia, and with the agreement and support of the Consejería de Cultura, Patrimonio Histórico, Educación y Juventud of the Cabildo Insular de Fuerteventura.

**Table 1:** Fossil samples used in this study, all from Fuerteventura (Canary Islands).

Sample	Description	Site	From calibrated Ages (AMS ^14^C)
CH559	Skull (1 individual)	Cueva del Llano	ca 800 BC [5]
CH560	1 half-mandible (1 individual)	Cueva del Llano	ca 800 BC [5]
CH475	3 half-mandibles pooled (3 individuals)	Montaña de la Arena	<1300 BC [6]
CH476	1 half-mandible (1 individual)	Montaña de la Arena	<1300 BC [6]

In Cueva del Llano, white specimens were chosen because of their colour that may indicate a possible younger age than brown coloured specimens. They had been collected directly from the youngest layer, and the dates published for the cave stratigraphy can be extrapolated to these specimens [5], [6]. An age of ca. 800 calibrated years BC can be assigned for Cueva del Llano specimens [5]. The bones from Montaña de la Arena are probably younger than 1300 calibrated years BC [6].

### Ancient DNA analysis

Paleogenetic work was carried out at PALGENE, the French National Platform of Paleogenetics (CNRS, ENS Lyon, France), following strict ancient DNA procedures [14], [15]. DNA was extracted from bone samples following the phenol–chloroform–isoamylalcohol method (described in [14], [16]) or the silica based-protocol detailed by [17]. Samples from non-relative species such as *Ursus spelaeus* (cave bear), *Canis familiaris* (dog) and *Crocuta spelaea* (cave hyena) were extracted at the same time to evidence any putative carrier-effect of the ancient DNA extracts [15], [16], [18].

**Figure 2:**
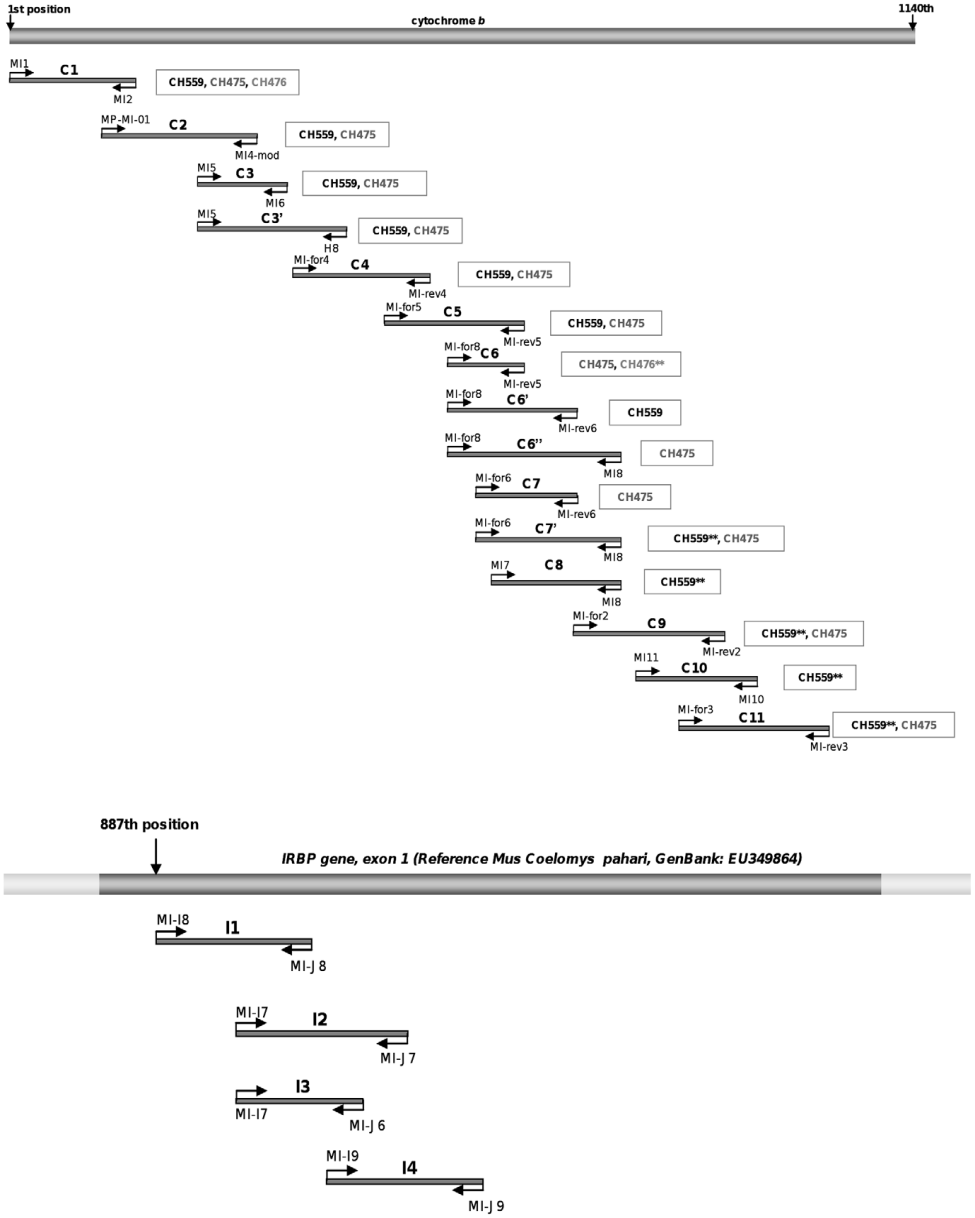
Map of the primer's locations on the two genes sequenced in this study, *cytb* and IRBP. IRBP primers are positioned on the reference sequence of *Mus Coelomys pahari* (GenBank accession number EU349864). For each *mt* fragment, samples that yielded successful amplifications are mentioned into boxes. ** indicates PCR attempt where numts were occasionally co-amplified.

15 short overlapping fragments (97–219 bp including primers) were targeted to obtain the nearly whole cytochrome *b* gene (*cytb*). 4 fragments were defined to cover 271 bp of the first exon of the gene encoding the Interphotoreceptor Retinoid Binding Protein (IRBP) (Figure 2). Primer sets (Table 2) were designed based on the alignment of extant murid species sequences available in GenBank. Generally, at least two independent PCR amplifications by fragment were performed in 25 µL reaction volumes containing 2.5 units of Perkin Elmer Gold *Taq* polymerase (Applied Biosystems), 1 mg/mL BSA (Roche, 20 mg/mL), 2 mM MgCl2, 250 µM of each dNTP, 0.5 µM of primers. For each independent PCR attempt, a range of dilutions was performed to find the best compromise between the inhibitor's concentration and the targeted DNA molecule concentration [19]. DNA was amplified with a 5 min activation step at 95°C followed by 55 to 60 cycles of denaturation (94°C, 30 s), annealing (see Table 2 for temperature, 30 s) and elongation (72°C, 45 s). Four independent blanks were carried out for each PCR attempt: i) an extraction blank to monitor exogenous contamination during extraction, ii) a PCR blank to control PCR products, iii) a PCR blank that remained opened during PCR to monitor aerosols during PCR preparation, and iv) a cross-contamination blank to control a putative carrier-effect of the ancient DNA extracts [15], [16]. Amplification products were systematically cloned using Topo TA Cloning for sequencing kit (Invitrogen) to check out artefactual substitutions due to post-mortem DNA decay. Clones of independent amplifications were sequenced to determine consensus sequences (Macrogen Seoul Korea; Genome Express, Grenoble; Cogenics).

**Table 2:** Primers and PCR cycling conditions used in this study.

Fragment	Position	Forward primer (5′→3′)	Reverse primer (5′→3′)	length (bp)	Tm (°C)
C1	1–159	MI1: ATGCAAACATCCGAAAAAC	MI2: GTATGGCTAGAAATAGGCCTG	159	54
C2	116–311	MP-MI-01: CTGCCTAATAGTACAAATT	MI4-mod: ATAGTATATTCCTCGTCCGAT	196	50
C3	237–349	MI5: CGATATATACACGCAAACGGAGC	MI6: ACTCCAATGTTTCATGTTC	113	54
C3′	237–424	MI5: CGATATATACACGCAAACGGAGC	H8: CCTCAGAATGATATTTGTCCTC	188	52
C4	357–529	MI-for4: CTATTCACAGTTATAGCTACGGC	MI-rev4: CGTGTTAGTGTGGCTTTRTCTACTG	173	52
C5	472–647	MI-for5: CAACCCTAGTAGAATGAATYTGAGG	MI-rev5: GACTGCGTCCGAGTTTAGGCCTG	176	52
C6	551–647	MI-for8: CCTACCATTTATAATTACAGC	MI-rev5: GACTGCGTCCGAGTTTAGGCCTG	97	52
C6′	551–714	MI-for8: CCTACCATTTATAATTACAGC	MI8: ATATAGTTGTCAGGGTCTCC	164	52
C6″	551–769	MI-for8: CCTACCATTTATAATTACAGC	MI-rev6: GAAATARTAGTATRATTAGGATTCC	219	52
C7	587–714	MI-for6: CCTACTATTTCTTCATGAAACCG	MI-rev6: GAAATARTAGTATRATTAGGATTCC	128	52
C7′	587–769	MI-for6: CCTACTATTTCTTCATGAAACCG	MI8: ATATAGTTGTCAGGGTCTCC	183	52
C8	607–769	MI-7: CAGGCTCAAACAACCCAACAGG	MI8: ATATAGTTGTCAGGGTCTCC	163	55
C9	710–900	MI-for2: ATTTCTTATAAYTCTAGTACT	MI-rev2: AAATTAGGATAGATAAAATTA	191	42
C10	789–941	MI11: ACCCCACCTCATATCAAACCAG	MI10: GCTGCGTTGTTTTGAGGTGTG	153	52
C11	843–1031	MI-for3: CGYTCCATYCCCAATAAACT	MI-rev3: TTCTACTGGTTGACTTCCAA	189	50
I1	887–1037	MI-I8: CTCCAGGACTATTACACATTAGTG	MI-J8: CCAGTAGCCTGGGATCCTCTG	151	52
I2	965–1131	MI-I7: GTCTCTGAAGAGGACCTGGTGAC	MI-J7: GCTTCTTCCTTGGGCACCTCRGG	167	52
I3	965–1087	MI-I7: GTCTCTGAAGAGGACCTGGTGAC	MI-J6: AGGCCCAGTCTCAGGTCTTGAGG	123	52
I4	1053–1204	MI-I9: CCAGAGACTCCTCCACAAGACC	MI-J9: CAAAGCGCAGGTAGCCCACATTGC	152	52

C1 to C11: Cytb fragments, I1 to I4: IRBP fragments.

IRBP reference sequence = *Mus Coelomys pahari*, GenBank: EU349864.

### Phylogenetic analyses

*Malpaisomys* sequences were added to a larger dataset including sequences of extant species representing 3 subfamilies of Muridae: Murinae (36 genera, 60 species), Deomyinae (4 species) and Gerbillinae (3 species). Gerbillinae were included since they were closely related to Deomyinae and Murinae [20], the two putative subfamilies to which *Malpaisomys* might belong [3], [11]. Species were selected in order to represent each tribe of Murinae *sensu* Lecompte *et al.*, 2008 [10] and depending on their availability in GenBank (Table S1). *Cytb* and IRBP sequences were aligned by eye and translated into peptide sequences to ensure sequence orthology. Congruence between mitochondrial (*mt*) and nuclear phylogenetic information was first assessed via phylogenetic reconstruction based on each independent gene. The two nucleotide datasets were then combined into a single one using SEAVIEW [21]. Thus, a total of 2,376 bp were considered in the subsequent phylogenetic analyses (992 bp of the *cytb* and 271 bp of the IRBP gene were obtained for *Malpaisomys*, the remaining part of the sequences was thus treated as missing data for this taxa). To be sure that missing data could not bias our results, phylogenetic reconstructions were re-run on the same datasets (*cytb*, IRBP and concatenated dataset) but limited to the data available for *Malpaisomys*.

Phylogenetic trees were reconstructed using two probabilistic approaches: maximum likelihood (ML) and Bayesian inferences (BI). The appropriate model of evolution was first determined for each gene and each codon position using corrected Akaike information criterion (AICc) and the Modeltest software [22]. The general time-reversible GTR+I+Γ model was selected for both *cytb* and IRBP partitions. Partitioned ML analysis was performed using RAxML 7.0.3 [23]. The GTR+Γ+I model (option –m GTRGAMMAI) was selected for the six partitions (option –q multipleModelFileName). Individual α-shape parameters, GTR-rates, proportion of invariable sites and base frequencies were estimated and optimized for each partition. Robustness of the tree was assessed using the default bootstrap procedure with 1,000 replications (option -# numberOfRuns) using the ML tree estimated with RAxML tree as starting tree (option –t) [24]. Bayesian analyses were performed using MrBayes v3.1 [25]. Four independent runs of 10,000,000 generations sampled every 100^th^ generation were performed applying independent GTR+I+Γ model of evolution to each partition (partitioning by gene and by codon position). A burn-in period of 2,500 trees (250,000 generations) was determined graphically using Tracer1.2 [26].

### Assessing the confidence of the tree selection

Likelihood-based tests of alternative topologies were achieved using Bayes factors (*i.e.* the ratio of marginal likelihoods of two competitive models, here two competitive topologies) following methods described in [27]. MrBayes [25] was used to compare the optimal topology (*i.e.* the MrBayes tree) to topologies constrained to contain alternative monophyletic group including *Malpaisomys* (*e.g. Malpaisomys* embraced in a monophyletic Arvicanthini group). The harmonic mean of the likelihood values sampled from the posterior distribution of the MrBayes analysis was considered as a rough estimate of the marginal likelihood of the hypothesis [28]. Harmonic mean of the log-likelihoods were calculated using the sump command in MrBayes, based on the pooled likelihood scores of the post–burn-in trees (burn-in = 2,500) from the replicate searches for each topology (two independent runs of 10,000,000 generations sampled every 100^th^ generation, applying independent GTR+I+Γ model of evolution to each partitions). When value of (2*ln*BF) was higher than 10, it was considered to be very strong evidence favouring one hypothesis (*i.e.* the topology with the highest likelihood score) over the other [29].

Shimodaira-Hasagawa (SH-) tests and Approximately Unbiased (AU-) tests were also performed to compare the best RAxML tree to alternative topologies. For a SH-test be accurate, the set of the compared topologies should necessarily contain every topology that can be possibly entertained as the true topology [30]. As the a priori selection of the topologies could be thorny, we decided to achieve AU-tests that relax this assumption. The site-wise lo-likelihoods of each tree was estimated from the concatenated dataset using PAUP [31] (Lscores command, options sitelikes, scorefile and SHtest = FullOpt) and applying a GTR+I+Γ model. Log-likelihoods of site-pattern trees were then used to calculate the p-values for the AU- and the SH- tests in CONSEL [32].

### Estimating dates of divergences

Divergence dates were estimated using the relaxed molecular clock approaches accounting for change in evolutionary rate over times, implemented in Multidivtime [33] and in BEAST version 1.6.1 [34], [35]. Fossil calibrations (FC) of node were set as hard bounds in Multidivtime, as soft bounds in BEAST (lognormal distribution with the median value equal to the mean of fossil calibration). We selected the three following fossil calibrations:

FC1: Following Steppan [36], we considered the detailed and well-calibrated fossil record from the Siwalik succession in Pakistan as an accurate depiction of murine history and *Progonomys* as either the Most Recent Common Ancestor (MRCA) of extant murines or a predecessor (12.3–8.1 Ma, [37]). *Antemus* which does not present the fully developed modern murine configuration (*i.e.* the synapomorphic triserial cusp arrangement of modern murines first seen in *Progonomys* but not in *Antemus*) predates the MRCA of murines [37]. Consequently we assigned the oldest record of *Progonomys* at 12.3 Ma [37] to the split between the tribe Phloemyini and the other tribes of Murinae. The conservative interval of 10.3–14.3 Ma was used in Multidivtime analysis.

FC2: Fossils of *Apodemus jeanteti* (7 Ma) and *Apodemus dominans* (7 Ma) were considered to be affiliated to *A. mystacinus* and *A. sylvaticus* respectively [38]. Consequently, we assigned a minimum age of 7 Ma to the split between *A. mystacinus* and *A. sylvaticus*.

FC3: We assigned a minimum age of 5.7 Ma to the split of Otomyini/Arvicanthi as the earliest record of the African Arvicanthini representative is dated between 5.7–5.9 Ma [39].

Even if soft bounds were set in BEAST, if there is no significant signal in the data, then the posteriors will be entirely affected by the priors. Consequently, the analyses were first performed omitting each of the calibration points in turn to assess whether the molecular dates are the result of the fossil constraints (*i.e.* if the same or similar divergence dates are always recovered) with BEAST and Multidivtime.

In Multidivtime, divergence times were estimated from the partitioned dataset (*cytb* and IRBP) for the RAxML topology following the documentation files written by Rutschmann [40] and using parameter estimates derived with PAML [41] as described by Yoder and Yang [42]. In BEAST, an uncorrelated lognormal model of evolution rate variation, a Yule speciation model for branching rates (species-level phylogeny), and a GTR+I+Γ nucleotide substitution model were implemented on the partitioned dataset (*cytb* and IRBP). Substitution and clock models were unlinked among partitions. The starting topology was the RaxML tree as in Multidivtime analysis. The analysis was run for 80 million generations and sampled every 1,000 generations. Convergence of the run as well as the effective sample size of each parameter trace was assessed using Tracer1.2 [26]. Posterior estimates and 95% Highest Posterior Density (HPD) limits of node heights of trees produced by BEAST were summarized using TreeAnnotator (burnin = 20,000), distributed in BEAST package.

## Results and Discussion

### Ancient DNA retrieval and sequence authentication

Sequences obtained in this study were deposited in EMBL with the following accession numbers JN418213-JN418217. For *mt* DNA, 992 bp of the *cytb* gene were determined for two samples (*i.e.* CH475 and CH559) using 12 to 15 overlapping fragments (Figure 2 and Table 3). Only two *mt* fragments (C1 and C6) were amplified for CH476 and none were obtained for CH560 (Table 3). Because *mt* DNA is present in higher copy number than nuclear DNA per cell, it is usually more difficult to obtain nuclear fragments than *mt* ones [43]. Indeed, only one sample, CH559, allowed the amplification of 4 overlapping fragments of the IRBP gene, *i.e.* 271 bp (Table 3). In summary, we were able to detect preserved DNA in 3 out of 4 samples analysed. Similar DNA preservation rates are reported in the literature for Canarian fossil specimens (*e.g. cytb* sequences obtained from four out of five specimens of the extinct seabird, *Puffinus olsoni*; no successful amplifications for four fossils of the dune shearwater, *Puffinus holeae* [44]). These moderate success rates are probably due to the warm climatic conditions that prevail in the Canary archipelago, known to be unfavourable for long-term DNA preservation.

**Table 3:** Positive PCRs, frequency and types of artefactual mutations per samples.

Sample	PCR fragment	Length (bp)	PCR	Clones	Transition				Transversion								%TS	% C*	%TV	*Numt*
					T→C	C→T	A→G	G→A	C→G	A→C	T→A	G→T	A→T	C→A	G→C	T→G				
**CH475**	C1	119	5 (2)	37		10	1	4	1								94%	88%	6%	
	C2	156	4 (2)	32	2	5					1						88%	63%	13%	
	C3	70	3 (1)	11													0%	0%	0%	3/3
	C3′	143	2(1)	16	1	7		1									100%	89%	0%	
	C4	125	4(2)	32		3		6									100%	100%	0%	
	C5	128	2 (1)	13	1	7	2									1	91%	64%	9%	
	C6	53	4 (3)	37	2	6		6									100%	86%	0%	
	C6′	178	2(1)	16		7	2	4									100%	85%	0%	
	C7	80	2 (1)	16	1	5		2									100%	88%	0%	
	C7′	140	4 (2)	31		9	1	3									100%	92%	0%	
	C9	149	4 (2)	31		11	1	2	2								88%	81%	13%	
	C11	149	4 (2)	31	1	6	2	1				2					83%	58%	17%	
**CH476**	C1	119	1 (1)	11			1	1									100%	50%	0%	
	C6	53	3 (1)	16		2											100%	100%	0%	1/3
**CH559**	C1	119	5 (3)	31	1	8											100%	89%	0%	
	C2	156	4 (2)	30	1	9	2	4			1						94%	76%	6%	
	C3	70	4 (2)	29	3	4		4						1			92%	67%	8%	
	C3′	143	4 (3)	25	6	13	3	4				2					93%	61%	7%	
	C4	125	2 (2)	16		2	1		2	1			1	1			38%	25%	63%	
	C5	128	2 (2)	15	3	3	2	1		1							90%	40%	10%	
	C6″	118	2 (2)	16	1			1									100%	50%	0%	
	C7′	140	2 (2)	9													0%	0%	0%	2/2
	C8	121	3 (2)	13	1												100%	0%	0%	1/3
	C9	149	2 (1)	10		2		4									100%	100%	0%	1/2
	C10	110	3 (1)	6				1									100%	100%	0%	2/3
	C11	149	2 (1)	10	1		1	4									100%	67%	0%	
**CH559**	I8 J8	106	3 (2)	19				1					1				50%	50%	50%	
	I7 J7	121	1 (1)	7													0%	0%	0%	
	I7 J6	73	1 (1)	7				1									100%	100%	0%	
	I9 J9	107	2 (2)	17	1	3		1									100%	80%	0%	

To compute the degradation frequencies, the number of changes between the consensus sequence and each clone were counted and added for all clones of the same sample. Only one change was taken into account in the case of a mutation occurring in several clones at the same place. Numbers in brackets indicate the number of independent PCR sessions. TS: transition, C*: deaminated cytosines, TV: Transversion.

When targeting *mt* DNA, numt amplifications are common and largely documented for rodents (*e.g.* in *Apodemus* [45]; *Calomys* [46]). If not detected, numts can lead to an incorrect interpretation of the data and to an inaccurate phylogenetic position of the analysed species [47]. In our study, overlapping fragments yielded an almost complete *cytb* sequence (992 bp) considered as the genuine *mt* copy. We also detected occasional amplifications of divergent sequences showing 5.4 to 20.9% of differences with this genuine sequence: a 97 pb fragment (including primers) for CH476 and 5 fragments of 153 to 189 bp for CH559 for a total length of 402 bp were concerned (Figure 2). At first sight, these sequences were considered as numts since they were obtained at a very low frequency compared to the “genuine” ones [43]. This observation is consistent with the fact that the number of *mt* genuine copies per cell is higher than the number of nuclear numt copies. However, we did not detect any stop codon or nucleotide insertion into these putative numts when translated into amino acids. The third position base composition of both kinds of fragments was also typical of the mammal *cytb* (*i.e.* A 39%, C 36%, G 3% and T 21%; [48]). Patterns of mutation rates at the codon positions (expected to be higher at the third positions, followed by first and then second positions for the genuine copy/expected to be less contrasted for the non-coding numt) were identical for all fragments considered. Both genuine and numt sequences, although different, gave similar percentages of identity when compared by BLAST tool to sequences available in GenBank (83% to 94% according to fragment considered). Because of the slower mutation rate in nuclear DNA, numts may exhibit much shorter branch lengths when included in phylogenetic reconstructions and may be placed at a basal position in the tree [43]. However, phylogenetic analyses performed either on what we thought to be the genuine *mt* sequence or the numt sequences didn't change the phylogenetic position of *Malpaisomys insularis* among rodents (data not shown). All previous observations seem to indicate that numt sequences amplified are *Malpaisomys insularis* - specific. In order to confirm our first conclusion about the “genuine” *mt* copy, we focused on the results obtained for the nuclear marker IRBP. For this nuclear gene, it was impossible to obtain a nuclear fragment longer than 123 bp for CH475 and CH476 specimens (no amplification out of the 22 attempts for the IRBP fragments whose length was comprised between 123 and 152 bp for CH475; no amplification out the 18 attempts for the same fragments for both CH476 and CH560). Consequently, nuclear fragments higher than 123 bp (corresponding to the smallest nuclear fragment we attempted here) don't seem to be preserved for these samples. The *mt* fragments obtained for them that are longer than 123 bp are thus likely to correspond to the genuine *mt* copy rather than to a numt copy. To reinforce this statement, CH475 and CH476 *mt* amplicons longer than 123 bp were indeed always homogeneous (*i.e.* only one type of sequence was retrieved). Using this rational, the longest *cytb* fragments obtained for CH475 and CH476 were used to distinguish between numt and true *cytb* copies in all *mt* amplicons. By this mean, we were able to definitively confirm our first identification of the genuine *mt* and the numt copies. Only the genuine *cytb* copy was considered in the further analyses.

To remove the artefactual mutations due to post-mortem DNA degradations, we cloned and sequenced at least two different amplicons for each PCR fragment and whenever possible two independent ones (*i.e.* PCRs performed at different days, Table 3). Fragment C1 was obtained only once for CH476 but was totally identical to the consensus sequence obtained for CH559 and CH475. Fragments I2 and I3 were also amplified once for CH559. But as they are largely overlapping (Figure 2), these two amplicons were considered as valid independent replicates. Ambiguities due to ancient DNA decay were alleviated in all consensus sequences performed for each sample, except three positions in the *cytb* fragment C11 of CH559 (R938, R941, R1000). The 2 independent positive amplifications yielded different residues at 3 positions: 1/A938/A941/A1000 and 2/G938/G941/G1000. These uncertainties are likely to correspond to G residues (see Data S1 for justification). Consensus sequence of *Malpaisomys* used in the subsequent phylogenetic analyses corresponds to the combination of the consensus sequences obtained for the 3 samples. To be conservative, we decided to keep the 3 positions unresolved for CH559 *cytb* consensus as undetermined positions (*i.e.* R) in the final consensus sequence. For the same reasons, two positions (36 and 44) in the IRBP fragment I4 obtained for CH559 were coded as undetermined (Y36 and Y44).

We argue that these *cytb* and IRBP sequences are authentic *Malpaisomys* sequences for the following reasons: (i) rodent samples were never introduced in the ancient DNA facilities before this analysis, (ii) the errors induced by DNA damages are perfectly congruent with the pattern generally observed for ancient DNA sequences (strong bias toward type 2 transition caused by cytosine deamination, see Table 3) [49], [50], (iii) extraction, PCR and aerosol controls performed during the same PCR sessions were negative, (iv) no PCR product was obtained when using *Malpaisomys* primers on bear and hyena co-extracted or co-amplified with rodent samples CH559, CH475 and CH476, suggesting that no carrier effect and thus no contamination occurred during the amplification process, (v) the sequences we obtained were identified as close to murid ones using a BLAST program but were never identical to any known murid sequences available in GenBank, (vi) independent analyses carried out on distinct samples by different manipulators, yielded the same sequences. All in all these points satisfy criteria of authentication for the ancient DNA work [51], [52].

### Phylogenetic position of *Malpaisomys insularis* based on molecular evidences

**Figure 3:**
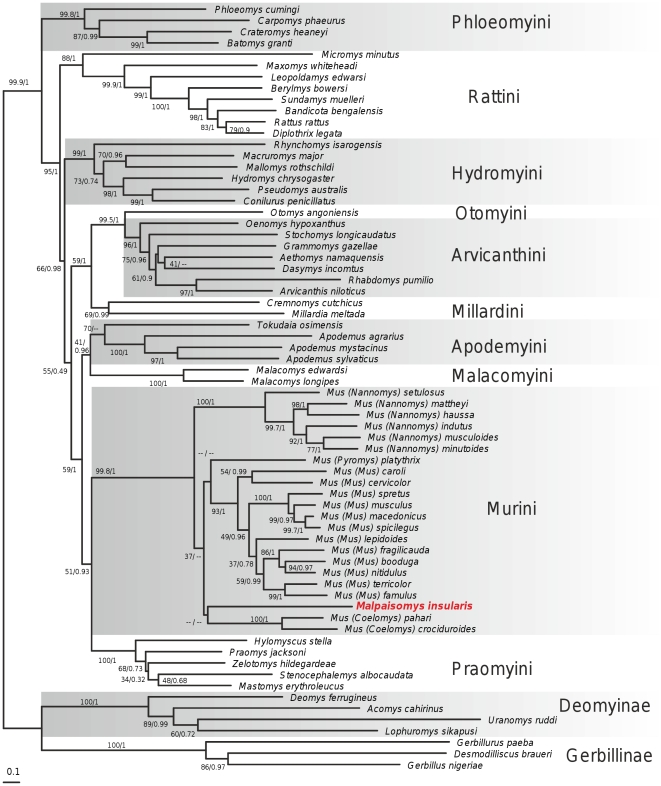
RAxML tree depicting the relationships of *Malpaisomys* within Muridae based on the analysis of the combined *cytb* and IRBP genes. ML and BI analyses of the dataset gave a similar topology. Numbers above the branches reflect support obtained from the analysis of the dataset following the two different reconstructions (BP _RAxML_/PP _MrBayes_. The symbol “–” indicates that phylogenetic relationships are not supported by one of the two analyses. Tribal arrangement following [10] is indicated on the right of the tree.

Analyses of the combined dataset (2,376 bp, 68 taxa) yielded the best ML topology presented in Figure 3. BI and ML trees were congruent except concerning the position of the Ryukyu Island endemic genus, *Tokudaia*, and some relationships among the tribes Arvicanthini and Murini. In the ML tree, *Tokudaia* appears as the sister taxon of the genus *Apodemus* with moderate support (70% BP) while it is grouped in sistership with the Malacomyini in the BI tree with low PP (0.54). This discrepancy is not surprising since the phylogenetic position of *Tokudaia* is known to have been problematic for decades: if a close relationship between *Apodemus* and *Tokudaia* was suggested on dental morphology, *e.g.* [53], molecular supporting data were only recently obtained [54], [55], [56]. Incongruence among the Murini and Arvicanthini tribes concerns only low support value branching patterns and are just inconclusive regarding these positions. The topology we obtained in this study for the Muridae is congruent with the topology obtained by Lecompte *et al.* [10] using a larger dataset but encompassing the same genes. The sub-families of Gerbillinae, Deomyinae, and Murinae appear as monophyletic groups with maximal supports (BP≥99.9%, PP = 1.00) and tribes recognized among the Murinae subfamily are well supported (BP≥88% except the Millardini tribe, 69% BP and the Apodemini tribe, 70% BP; PP≥0.99 for all the tribes except the Apodemini grouping that is not supported in the BI tree).

In this tree, *Malpaisomys insularis* is placed without ambiguity among the Murini tribe (monophyly of this clade being supported with high supports, 99.8% BP and 1.00 PP). Phylogenetic reconstructions based on each independent gene gave exactly the same result with BP = 92 and PP = 1.00 for the *cytb* gene and BP = 98 and PP = 1.00 for the IRBP gene (Figures S1 and S2). Phylogenetic relationships among the Murini, *i.e.* between the four subgenera of *Mus* (*Nannomys, Coelomys, Pyromys* and *Mus*), and *Malpaisomys*, are poorly resolved based on the combined dataset (Figure 3). The lava mouse was placed as the sister taxon of *Mus pahari* and *Mus crociduroides* in the best ML tree and the bootstrap analysis supports an association with *Mus platythrix* but with weak support (BP: 31%). Bayesian tree grouped *Malpaisomys* with *Mus platythrix* but with very low PP (0.42). Even if the amount of missing data was demonstrated to have a limited effect on the final topologies and their associated measures of support [57], we re-analyzed the total or gene-by-gene datasets, limiting the analyses to regions that include lava mouse data (992 bp, 452 informative characters, 68 taxa for the *cytb*; 274 bp, 95 informative characters, 65 taxa for the IRBP; 1266 bp, 543 informative characters, 68 taxa for the combined dataset), to test whether a similar placement within Murini is recovered. These additional analyses without missing data grouped *Malpaisomys* with the genus *Mus* with moderate to strong support (BP = 67, PP = 1.00 for the IRBP; BP = 81, PP = 1.00 for the *cytb*; BP = 98, PP = 1.00 for the combined dataset, Figure S3). In any cases, the relationships among the Murini, are once again poorly supported. Resolving the relationships among the tribe Murini is known to represent a real challenge and similar inconclusive results have been reported in previous studies (*e.g.* [58], [59], [60]).

**Figure 4:**
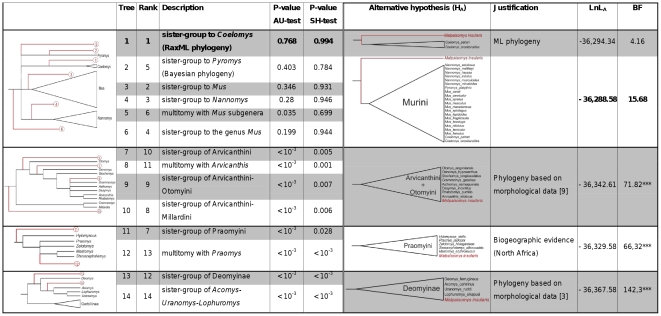
Tests of alternative topologies concerning *Malpaisomys* phylogenetic position among Muridae (SH-, AU-tests and Bayes Factors). The best topology is indicated in bold. BF values higher than 10 were considered as a very strong evidence favouring one hypothesis (i.e. the topology with the highest likelihood score) over the other [29]. *** indicates BF factors where the BI tree topology (LnL_0_ = −36,296.42) is significantly better than the tested alternative topology.

Whatever its position inside the mouse clade, tests of alternative topologies indicate without any ambiguity that *Malpaisomys* belongs to the Murini tribe (Figure 4). Indeed, alternative hypotheses where *Malpaisomys* does not belong to the monophyletic group of the Murini (*i.e.* Arvicanthini, Deomyinae and Praomyini hypotheses) are strongly rejected whatever the methods or the statistics (p value<5% (SH-test) and 1<‰ (AU-test)/BF>66). On the contrary, alternative branching patterns of *Malpaisomys* among the Murini do not appear as significantly different considering SH-, AU-tests and BF (BF _RAxML tree versus MrBayes tree_ = 4.16<10). However, BF gave positive evidence in favour of trees where *Malpaisomys* is placed at the base of the Murini, as currently defined, when the Bayesian tree or the RAxML tree represent the null hypothesis (BF = 15.68 and 11.52, respectively).

### A re-examination of morphological characters in the light of molecular findings

A close phylogenetic affinity between mice (*i.e.* the members of the genus *Mus*) and *Malpaisomys* has never been envisaged by studies based on morphological characters ([3], [12], [13] and see [9]). How to interpret morphological characters and singularities of the lava mouse under the light of molecular conclusions?

**Figure 5:**
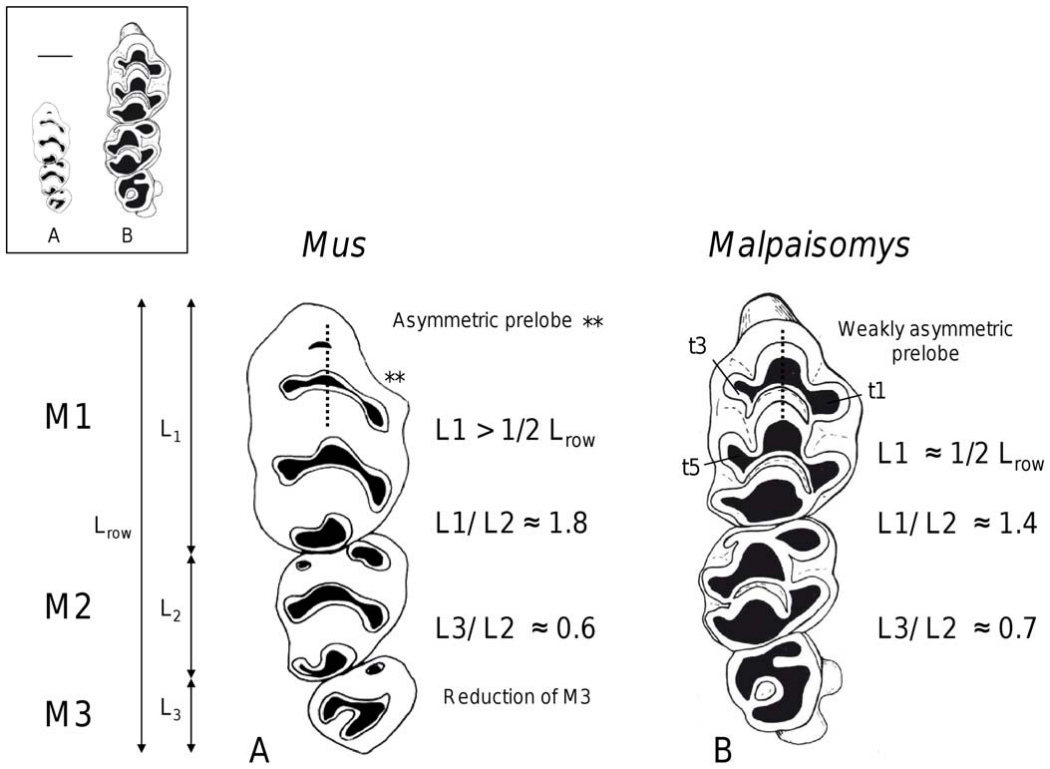
Upper right tooth rows. (A) *Mus*, (B) holotype of *Malpaisomys insularis* (Fig. 3 in [3]). In caption, scale is given by the horizontal bar (1 mm).

Molar characters used to recognize mice are: i) the asymmetry of the anterior part (prelobe) of the upper first molar consecutive to the distal position of the lingual cusp t1, ii) the relative elongation of the first upper molar and the reduction of the third upper one measured by the ratio of their length on the length of the second molar (L_M1_/L_M2_ and L_M3_/L_M2_) [53], and iii) the length of the first upper molar being greater than half of the tooth row [61] (Figure 5). One of the earliest representatives of the genus *Mus* such as the Mio-Pliocene species *Mus ique* described from Morocco [62] already has these characters. Another *Mus* of the Late Miocene, *Mus auctor* from the Siwaliks already exhibits the asymmetric outline of the anterior part of the first upper molar [63]. Tooth variability is known to be important between species of mice and up to date is still not explicitly described despite the fact that the lab mouse is one of the most studied mammals. But even if we consider the latter argument, the lava mouse clearly does not fit with mice characters. In *Malpaisomys*, the ratio of upper M1 length on half the total tooth row length approximates 1 (while it is higher than 1 for mice), and ratios L_M1_/L_M2_ and L_M3_/L_M2_ are respectively ca 1.4 and 0.7, while in *Mus ique* for example, values are 1.8 and 0.6 (Figure 5).

Moreover, the size of *Malpaisomys* was estimated to be comparable with the Broad-toothed Field Mouse, *Apodemus mystacinus* (*i.e.* about 110 mm head and body length based on reconstruction of the skeleton) and its body weight at about 40 g [64]. This weight represents more than twice the weight of a house mouse. However, if mice are rather small [65], insular species are expected to be large compared to their continental relatives. This was indeed the case for the extinct species of mouse found in Crete, *Mus minotaurus* [66], [67]. However, other characters of this species are those of mice, as for example, the ratio of M1 length on half the total tooth row length higher than 1 (Figure 5).

In order to reconcile molecular and morphological data, one hypothesis would be to consider *Malpaisomys* as a giant mouse according to the rather high frequency of the trend towards a larger size that affects small mammals that settled on islands [68], [69]. Size increase has been reported for the house mouse which settled on the Canary Islands very recently [5], [70]. If *Malpaisomys* was an insular lineage of mouse, its evolution was characterized by a widening of the molars that introduced a strong change in first upper molar outline. This change could be related to a shift in diet [71]. In correlation with this widening, cusps became more voluminous, and small accessory structures appeared in relation with cusps t1, t3 that are part of the prelobe, and cusp t5 of the first upper molar, or in the anterior side of the lower first molar (Figure 5). Changes also affected the skull of *Malpaisomys*: its skull is narrow at the level of the interorbital breadth [12], a character unknown in *Mus*. Such a character is related to a peculiar position of the eyes that may be adaptive as it allows a better protection from predatory birds, the eastern Canary Islands being characterized by open environments and great expanses of recent lava fields (“malpaíses”) covered by scarce vegetation [54].

Presently, molecular data indicate that the lineage of *Malpaisomys* split at a similar time as the genus *Mus* radiation but the exact order of lineage origins remains impossible to ascertain. This raises the following question: did the lineage of *Malpaisomys* split before the typical genus *Mus* dental pattern differentiated or not?

### Molecular dating

**Figure 6:**
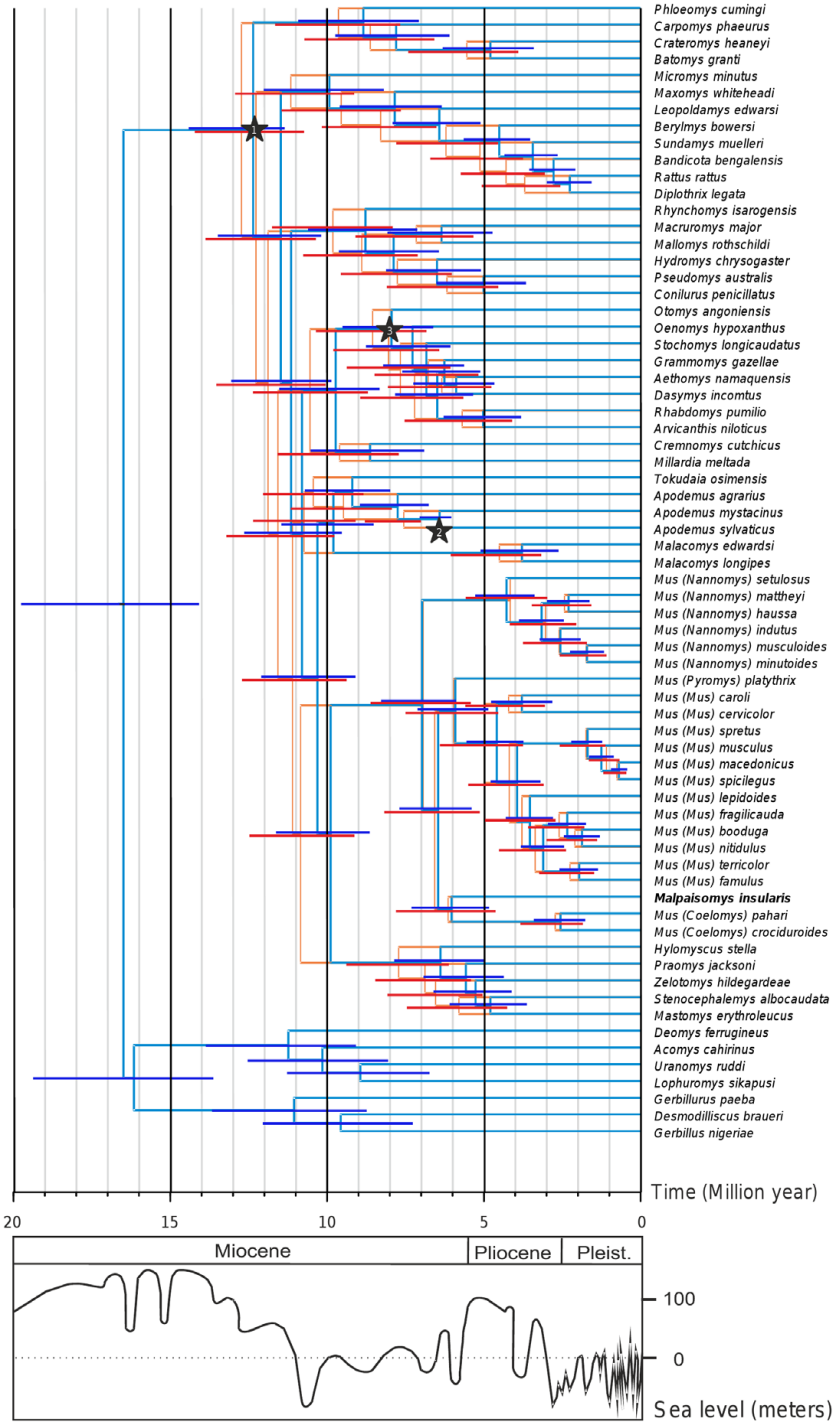
Divergence time estimates for Muridae. The chronograms were obtained under a Bayesian non-autocorrelated rates relaxed clock model using BEAST (in blue) and an autocorrelated model using Multidivtime (in orange) and applied to the combined *cytb*/IRBP dataset. 95% credibility intervals are drawn on nodes. The three fossil calibration points are indicated by a star. Variation of sea level is indicated below [84].

The chronograms derived from the BEAST and Multidivtime analyses are presented in Figure 6. Estimated ages and 95% credibility intervals (95% CI) of notable nodes are detailed in Table 4. Two independent runs of Multidivtime gave the same results. On the whole, the estimations of Multidivtime are slightly older that the ones obtained from the BEAST analysis, but in most cases, 95% CI are extensively overlapping and are of the same order (Figure 6). The estimations are particularly congruent among the Murini including *Malpaisomys* and for the deepest nodes of the tree while the most important discrepancies between the two methods are found among the Rattini (Figure 6). Different clock methods are known to lead to disparate results due to their inherent handling of the rate heterogeneity across lineages (*i.e.* random or autocorrelated lineage rates) [72]. When the lineage rate assumption is violated, relaxed-clock methods were proven to produce biased estimated credibility intervals [72]. This would explain that a specific evolutionary rate could characterize the Rattini tribe and account for the slight disparities between the two dating methods. Nonetheless, estimations and the 95% CI for the three calibration points are congruent with the paleontological evidence (Table 4). We re-ran the analyses, omitting each of the calibration points in turn to assess whether the molecular dates are the result of the fossil constraints and we obtained similar estimates (slightly older without FC1, younger without FC2 and FC3) (Table S2).

**Table 4:** Age estimates (Ma) using BEAST and Multidivtime methods.

Node	Paleontological Evidence	BEAST		Multidivtime	
		Node age	Height 95% HPD	Node age	95% CI
Murinae/Gerbillinae & Deomyinae		16.46	14.08–19.76	NA	NA
FC1: Phloeomyini/other Murinae	*Progonomys*: 12.3 Ma [37]	12.32	11.35–14.41	12.73	10.73–14.21
FC2: *Apodemus mystacinus/A. sylvaticus*	*Apodemus jeanteti* and *A. dominans:* 7 Ma [38]	6.38	6.04–7.061	7.55	7.02–8.80
FC3: Otomyini/Arvicanthini	Oldest Arvicanthini: 5.7 Ma [39]	7.91	6.62–9.51	8.54	6.83–10.35
Murini/Praomyini		9.85	8.64–11.63	10.84	9.12–12.46
Murini radiation		6.94	5.87–8.27	6.93	5.41–8.64
Praomyini		6.35	5.00–7.86	7.71	6.12–9.38

HPD for Highest Posterior Density, CI for Credibility Interval.

The split of the main lineages of Murinae took place between 12.3 and 9.8 Ma (BEAST)/12.7 and 10.8 Ma (Multidivtime). These estimates are similar to the ones obtained by Lecompte *et al.* [10], slightly older than the ones reported by Rowe *et al.* [54], and much younger than the ones found in Jansa *et al.* [73]. The main difference between our study and the one undertaken by Rowe *et al.* [74] corresponds to the addition of a third calibration interval (*i.e.* the *Apodemus mystacinus-A. sylvaticus* divergence). When this constraint is removed from the Multidivtime analysis, our estimates converged to the divergence-dates obtained by Rowe *et al.* [74] (Table S2). With respect to the study of Jansa et al. [70], the differences are probably not only due to sampling different taxa but also to the use of calibration points based on much older splits in the mammal lineage (overestimation of the shallowest nodes in the tree because of too ancient calibration points compared to these nodes [72]). Our molecular estimate of the age at which occurred the split of the main lineages of Murinae fits completely with the paleontological evidence, which indicates that the different modern lineages probably arose around 11 Ma. At that time, the first *Progonomys* and *Karnimata* are reported outside the Siwalik region, in Eurasia and Africa [75], [76], [77] and the modern lineages such as the *Apodemus* lineage arose in Europe [38].

The tribe Murini diverged from the Praomyini at 9.85 Ma (BEAST)/10.84 Ma (Multidivtime) with an overlapping CI interval of 12.46 to 8.64 Ma. The divergence of the different Murini subgenera including *Malpaisomys* took place at 6.94 (BEAST)/6.93 (Multidivdime) (CI = 8.3 −5.9 Ma (BEAST)/8.6 −5.4 Ma (Multidivtime)). We are confident in these estimates because they are highly congruent between the two approaches but also with previous molecular studies [58], [59] and with the oldest Murini representatives found in the fossil record of the Siwalik: *Mus auctor* (6.4 My) and *Mus* sp. (7.3 Ma) [37]. Consequently, the colonization of the Canary Islands could have occurred anytime after 6.9 Ma. However, since there is no paleontological evidence for the presence of *Malpaisomys* or its predecessor on the Canary Islands or on the continent previous to the Pleistocene, it is difficult to propose a date of arrival and a place for the origin of the lava mouse (Spain or Morocco). As the oldest *Malpaisomys* fossils are ca. 30,000 years old [8], its first occurrence predates the first human settlement (between 2,500 and 2,000 years ago, [6], [78], [79]). Thus, the hypothesis that a North African ancestor reached the eastern Canary Islands after 6.9 Ma ago during a major sea level regression via natural rafts is likely. Further phylogenetic investigations are now needed to corroborate this hypothesis and to determine whether the colonization of the Canary archipelago by terrestrial mammals corresponded to a single event or not. Investigating the phylogeny of the two other endemic extinct rodents, *Canariomys*, could also shed new light on this question.

### Conclusion

For the first time ancient DNA sequences show that *Malpaisomys* was more closely related to the genus *Mus* than to any other Murinae, a hypothesis that has never been investigated with morphological data. Further investigations are now needed to solve the inter-specific relationships among the *Malpaisomys*/mouse species clade. Such results will help to clarify whether *Malpaisomys* is embedded among the genus *Mus* and will have taxonomic implications for the whole group. Indeed, they will help to decipher if the mouse subgenera as currently defined (*i.e. Mus, Ceolomys, Nannomys* and *Pyromys*) [9] should be elevated to the rank of genus as already suggested [80], [81], [82]. They will also help to refine the timing of the colonization of the Canaries by the *Malpaisomys* ancestor and to elucidate the route taken to reach the archipelago.

## Supporting Information

Figure S1 **RAxML tree depicting the relationships of** ***Malpaisomys*** **within Muridae based on the analysis of the** ***cytb*** **gene.** Numbers above the branches reflect supports obtained from the analysis of the dataset following the two different reconstructions (BP _RAxML_/PP _MrBayes_.) The symbol “–” indicates that phylogenetic relationships are not supported by one of the two analyses. Numbers highlighted in bold and in grey indicate nodes which are respectively congruent and incongruent with the tree obtained based on the complete dataset (Figure 1).
(TIF)

Figure S2 **RAxML tree depicting the relationships of** ***Malpaisomys*** **within Muridae based on the analysis of the IRBP gene.** Numbers above the branches reflect support obtained from the analysis of the dataset following the two different reconstructions (BP _RAxML_/PP _MrBayes_.) The symbol “–” indicates that phylogenetic relationships are not supported by one of the two analyses. Numbers highlighted in bold and in grey indicate nodes which are respectively congruent and incongruent with the tree obtained based on the complete dataset (Figure 1).
(TIF)

Figure S3 **RAxML tree depicting the relationships of** ***Malpaisomys*** **within Muridae based on the analysis of the combined** ***cytb*** **and IRBP genes without the sites missing for** ***Malpaisomys*** **.** Numbers above the branches reflect support obtained from the analysis of the dataset following the two different reconstructions (BP _RAxML_/PP _MrBayes_.) The symbol “–” indicates that phylogenetic relationships are not supported by one of the two analyses. Numbers highlighted in bold and in grey indicate nodes which are respectively congruent and incongruent with the tree obtained based on the complete dataset (Figure 1).
(TIFF)

Table S1 **Sequence dataset extracted from GenBank and used in this study.** The tribal arrangement follows Lecompte's nomenclature [10].
(DOC)

Table S2 **Age estimates (Ma) using BEAST and Multidivtime softwares and omitting one of the three calibration points in turn.** HPD for Highest Posterior Density, CI for Credibility Interval, NA for Not Available.
(DOC)

Data S1 **Ancient DNA degradation for CH559 sample.**
(DOC)
