# Supplementary figures and images for: Paleogenetic Analyses Reveal Unsuspected Phylogenetic Affinities between Mice and the Extinct Malpaisomys insularis, an Endemic Rodent of the Canaries
Source: PLoS One. 2012 Feb 21;7(2):e31123. doi: 10.1371/journal.pone.0031123 (PMC3283599; doi:10.1371/journal.pone.0031123)

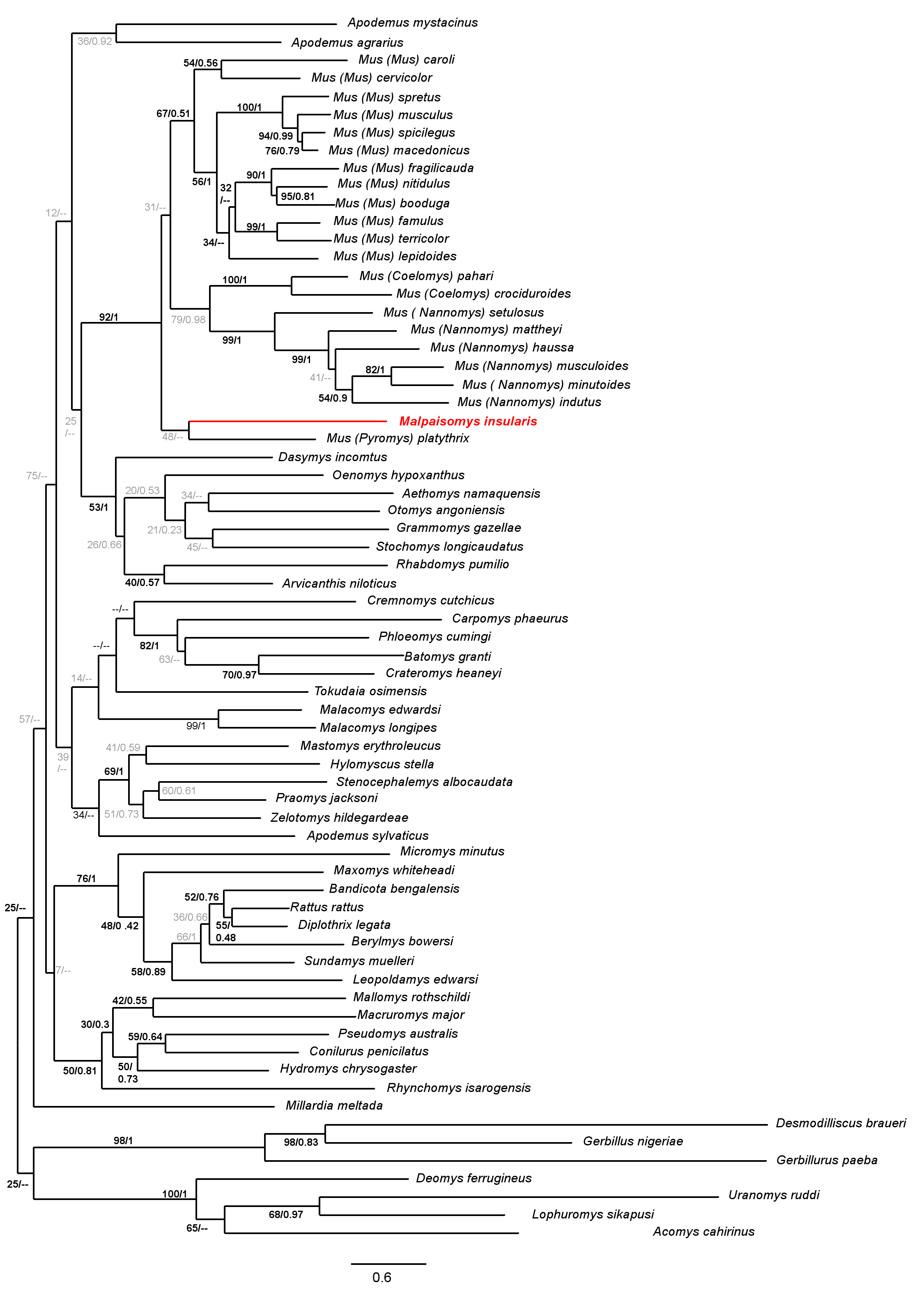

Supplement: Figure S1 — RAxML tree depicting the relationships of Malpaisomys within Muridae based on the analysis of the cytb gene. Numbers above the branches reflect supports obtained from the analysis of the dataset following the two different reconstructions (BP RAxML/PP MrBayes.) The symbol “–” indicates that phylogenetic relationships are not supported by one of the two analyses. Numbers highlighted in bold and in grey indicate nodes which are respectively congruent and incongruent with the tree obtained based on the complete dataset (Figure 1). (TIF) [file pone.0031123.s001.tif]

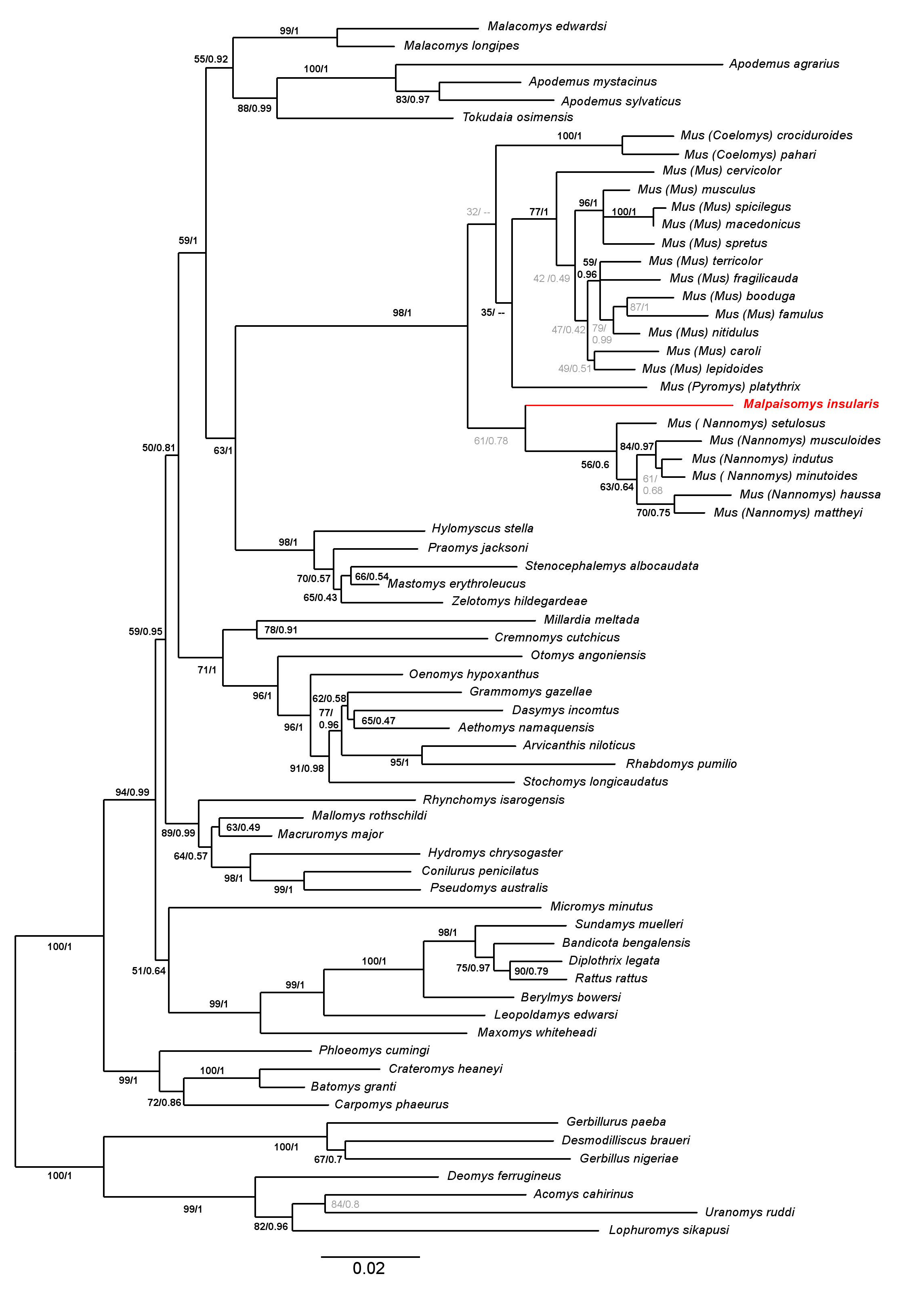

Supplement: Figure S2 — RAxML tree depicting the relationships of Malpaisomys within Muridae based on the analysis of the IRBP gene. Numbers above the branches reflect support obtained from the analysis of the dataset following the two different reconstructions (BP RAxML/PP MrBayes.) The symbol “–” indicates that phylogenetic relationships are not supported by one of the two analyses. Numbers highlighted in bold and in grey indicate nodes which are respectively congruent and incongruent with the tree obtained based on the complete dataset (Figure 1). (TIF) [file pone.0031123.s002.tif]

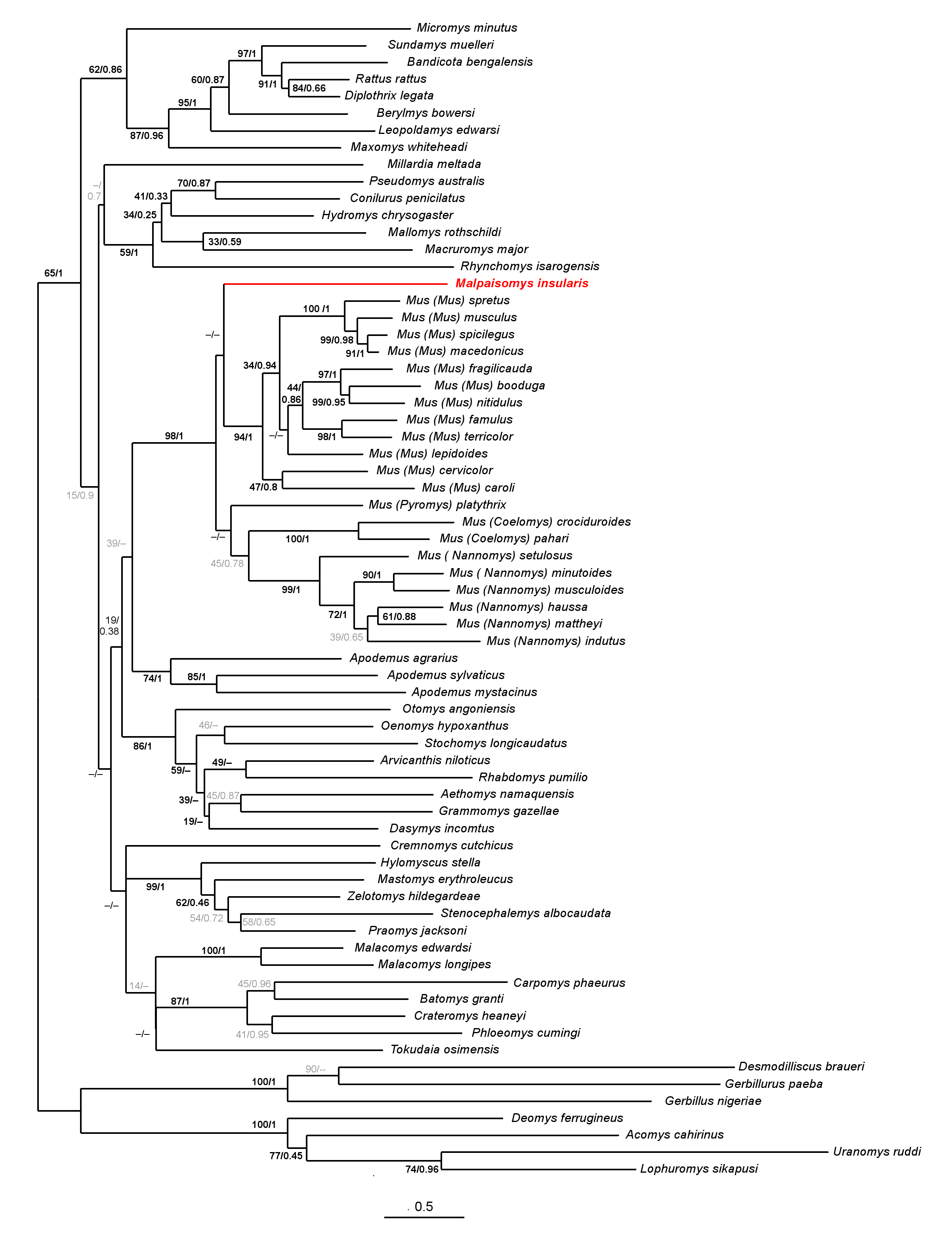

Supplement: Figure S3 — RAxML tree depicting the relationships of Malpaisomys within Muridae based on the analysis of the combined cytb and IRBP genes without the sites missing for Malpaisomys . Numbers above the branches reflect support obtained from the analysis of the dataset following the two different reconstructions (BP RAxML/PP MrBayes.) The symbol “–” indicates that phylogenetic relationships are not supported by one of the two analyses. Numbers highlighted in bold and in grey indicate nodes which are respectively congruent and incongruent with the tree obtained based on the complete dataset (Figure 1). (TIFF) [file pone.0031123.s003.tif]
