# Supplementary material for: Paleogenetic Analyses Reveal Unsuspected Phylogenetic Affinities between Mice and the Extinct Malpaisomys insularis, an Endemic Rodent of the Canaries
Source: PLoS One. 2012 Feb 21;7(2):e31123. doi: 10.1371/journal.pone.0031123 (PMC3283599; doi:10.1371/journal.pone.0031123)
Supplement: Table S1 — Sequence dataset extracted from GenBank and used in this study. The tribal arrangement follows Lecompte's nomenclature [10]. (DOC) [file pone.0031123.s004.doc]

.

|  | **Species** | ***cytb*** | ***IRBP*** |
| --- | --- | --- | --- |
| **Murinae** |  |  |  |
|  | **Phloeomyini** |  |  |
|  | *Batomys granti* | AY324458 | DQ191496 |
|  | *Carpomys phaeureus* | DQ191475 | DQ191501 |
|  | *Crateromys heaneyi* | DQ191476 | DQ191505 |
|  | *Phloeomys cumingi* | DQ191484 | AY326103 |
|  | **Rattini** |  |  |
|  | *Bandicota bengalensis* | AM408340 | AM408331 |
|  | *Berylmys bowersi* | AM408337 | AM407896 |
|  | *Diplothrix legata* | AB033696 | AB033706 |
|  | *Leopoldamys edwardsi* | AJ698881 | AJ698897 |
|  | *Maxomys whiteheadi* | DQ191481 | DQ191510 |
|  | *Micromys minutus* | AB033697 | AB033710 |
|  | *Sundamys muelleri* | AM408340 | AY326111 |
|  | *Rattus rattus* | AB033702 | AM408328 |
|  | **Hydromyini** |  |  |
|  | *Conilurus penicillatus* | AM910935 | AM910938 |
|  | *Hydromys chrysogaster* | AM408339 | AM408319 |
|  | *Mallomys rothschildi* | EU349758 | EU349854 |
|  | *Macruromys major* | EU349756 | EU349853 |
|  | *Pseudomys australis* | AM910936 | AM910939 |
|  | *Rhynchomys isarogensis* | AY324462 | AY326108 |
|  | **Arvicanthini** |  |  |
|  | *Arvicanthis niloticus* | AF004569 | DQ022386 |
|  | *Dasymys incomtus* | AF141217 | EU292143 |
|  | *Grammomys macmillani (=gazellae)* | AM408345 | AM408329 |
|  | *Micaelamys namaquensis* | AF141215 | AM408330 |
|  | *Oenomys hypoxanthus* | AM408342 | AM408324 |
|  | *Rhabdomys pumilio* | AF141214 | AY326106 |
|  | *Stochomys longicaudatus* | EU292149 | EU292147 |
|  | **Otomyini** |  |  |
|  | *Otomys angoniensis* | AM408343 | AM408325 |
|  | **Millardini** |  |  |
|  | *Cremnomys cutchicus* | DQ022381 | DQ022384 |
|  | *Millardia meltada* | AF141221 | AM408322 |
|  | **Apodemini** |  |  |
|  | *Apodemus agrarius* | AB032851 | AB032858 |
|  | *Apodemus mystacinus* | AF159394 | AB303229 |
|  | *Apodemus sylvaticus* | AB033695 | AB032863 |
|  | *Tokudaia osimensis* | AB029429 | AB033712 |
|  | **Malacomyini** |  |  |
|  | *Malacomys longipes* | AM408341 | DQ022393 |
|  | *Malacomys edwardsi* | DQ022379 | DQ022392 |
|  | **Praomyini** |  |  |
|  | *Mastomys erythroleucus* | AF518338 | AM408335 |
|  | *Praomys jacksoni* | AF518361 | AM408326 |
|  | *Hylomyscus stella* | AF518331 | AM408320 |
|  | *Stenocephalemys albocaudata* | AF518370 | DQ022414 |
|  | *Zelotomys hildegardeae* | AF518375 | DQ022396 |
|  | **Murini** |  |  |
|  | *Mus (Coelomys) crociduroides* | AJ698878 | AJ698894 |
|  | *Mus (Coelomys) pahari* | AY057814 | AJ698893 |
|  | *Mus (Mus) booduga* | AB125761 | AB125796 |
|  | *Mus (Mus) caroli* | AB253438 | AJ698885 |
|  | *Mus (Mus) cervicolor* | AB125766 | AJ698886 |
|  | *Mus (Mus) famulus* | AJ698872 | AJ698884 |
|  | *Mus (Mus) fragilicauda* | AB125780 | AB125814 |
|  | *Mus (Mus) lepidoides* | AB262414 | AB262416 |
|  | *Mus (Mus) macedonicus* | AB125770 | AB125805 |
|  | *Mus (Mus) musculus* | AB205275 | AB125808 |
|  | *Mus (Mus) nitidulus* | AB262422 | AB262424 |
|  | *Mus (Mus) terricolor* | AB125778 | AB125810 |
|  | *Mus (Mus) spicilegus* | AB125775 | AJ698882 |
|  | *Mus (Mus) spretus* | AB033700 | AJ698883 |
|  | *Mus (Nannomys) minutoides* | AJ875076 | AJ875086 |
|  | *Mus (Nannomys) musculoides* | AJ698875 | AJ698890 |
|  | *Mus (Nannomys) mattheyi* | AJ698876 | AJ698889 |
|  | *Mus (Nannomys) haussa* | AJ698877 | AJ698891 |
|  | *Mus (Nannomys) setulosus* | AJ875083 | AJ875088 |
|  | *Mus (Nannomys) indutus* | AJ698874 | AJ698892 |
|  | *Mus (Pyromys) platythrix* | AJ698880 | AJ698895 |
| **Deomyinae** |  |  |  |
|  | *Acomys cahirinus* | AJ233953 | AJ698898 |
|  | *Deomys ferrugineus* | FJ415478 | AY326084 |
|  | *Lophuromys sikapusi* | AJ012023 | AJ698899 |
|  | *Uranomys ruddi* | HM635858 | EU360812 |
| **Gerbillinae** |  |  |  |
|  | *Desmodilliscus braueri* | AJ851273 | FN357289 |
|  | *Gerbillurus paeba* | AJ430557 | AM910941 |
|  | *Gerbillus nigeriae* | AJ430555 | AM408333 |
