# Supplementary material for: Paleogenetic Analyses Reveal Unsuspected Phylogenetic Affinities between Mice and the Extinct Malpaisomys insularis, an Endemic Rodent of the Canaries
Source: PLoS One. 2012 Feb 21;7(2):e31123. doi: 10.1371/journal.pone.0031123 (PMC3283599; doi:10.1371/journal.pone.0031123)
Supplement: Table S2 — Age estimates (Ma) using BEAST and Multidivtime softwares and omitting one of the three calibration points in turn. HPD for Highest Posterior Density, CI for Credibility Interval, NA for Not Available. (DOC) [file pone.0031123.s005.doc]

|  | Without FC1 | | Without FC2 | | Without FC3 | |
| --- | --- | --- | --- | --- | --- | --- |
| Node | BEAST | Multidivtime | BEAST | Multidivtime | BEAST | Multidivtime |
|  | Age (Height 95% HPD) | Age (95 % CI) | Age (Height 95% HPD) | Age (95 % CI) | Age (Height 95% HPD) | Age (95 % CI) |
| Murinae / Gerbillinae & Deomyinae | 17.75 (13.69‑22.9) | NA | 15.73 (13.61‑18.43) | NA | 15.84 (13.76‑18.46) | NA |
| FC1: Phloeomyini /other Murinae | 13.38 (10.52‑17.16) | 13.86 (10.79‑18.72) | 11.88 (11.33‑13.35) | 11.56 (10.34‑13.86) | 11.92 (11.34‑13.38) | 12.72 (10.77‑14.2) |
| FC2: *Apodemus mystacinus /A. sylvaticus* | 6.46 (6.03‑7.54) | 7.89 (7.02‑10.25) | 5.31 (3.95‑6.73) | 5.19 (4.33‑7.91) | 5.32 (4.13‑6.6) | 7.56 (7.02‑8.8) |
| FC3: Otomyini / Arvicanthini | 8.45 (6.47‑10.86) | 9.25 (6.9‑12.76) | 7.47 (6.32‑8.88) | 7.64 (6.16‑9.66) | 7.67 (6.53‑8.96) | 8.54 (6.85‑10.32) |
| Murini / Praomyini | 10.56 8.37‑13.48) | 11.72 (9.15‑15.71) | 9.34 (8.15‑10.78) | 9.64 (8.16‑11.79) | 9.39 (8.27‑10.74) | 10.84 (9.17‑12.45) |
| Murini radiation | 7.42 (5.77‑9.53) | 7.48 (5.5‑10.43) | 6.56 (5.59‑7.69) | 6.16 (4.79‑8) | 6.61 (5.69‑7.71) | 6.95 (5.43‑8.66) |
| Praomyini | 6.75 (4.99‑8.95) | 8.36 (6.24‑11.49) | 5.96 (4.70‑7.33) | 6.84 (5.44‑8.75) | 6.03 (4.80‑7.39) | 7.72 (6.18‑9.38) |
