# Supplementary material for: Paleogenetic Analyses Reveal Unsuspected Phylogenetic Affinities between Mice and the Extinct Malpaisomys insularis, an Endemic Rodent of the Canaries
Source: PLoS One. 2012 Feb 21;7(2):e31123. doi: 10.1371/journal.pone.0031123 (PMC3283599; doi:10.1371/journal.pone.0031123)
Supplement: Data S1 — Ancient DNA degradation for CH559 sample. (DOC) [file pone.0031123.s006.doc]

Ambiguities due to ancient DNA decay were alleviated in all consensus sequences performed for each sample, excepted three positions in the *cytb* fragment C11 of CH559 (R938, R941, R1000). The 2 independent positive amplifications yielded different residues at 3 positions: 1/ A938/A941/A1000 and 2/ G938/G941/G1000. These uncertainties likely correspond to G residues because: 1) C/G →T/A changes due to deamination of C residues are frequent in ancient DNA templates [45,46] (see Table 4); 2) *cytb* consensus sequences of the different *Malpaisomys* specimens were 100% identical excepted at these undetermined position. CH475 consensus sequence (resulting of the analysis of four amplicons and two independent PCR sessions) exhibits a G at these 3 positions; 3) all the Murinae representatives of our dataset harbour a G residue at least for one of these site (G938). This character is probably shared by all the Murinae and outgroups representatives and thus by *Malpaisomys*; and 4) when amino acids are considered, A938 and A941 lead to His and Asn, while G938 and G941 yield to Arg and Ser. These two last amino acids, Arg and Ser, are conserved in Murinae but also in more distant mammals such as in *Canis*, *Bos* and *Lepus*.
